# Supplementary material for: Aβ receptors specifically recognize molecular features displayed by fibril ends and neurotoxic oligomers
Source: Nat Commun. 2021 Jun 8;12:3451. doi: 10.1038/s41467-021-23507-z (PMC8187732; doi:10.1038/s41467-021-23507-z)
Supplement: Supplementary file 1 — Supplementary Information [file 41467_2021_23507_MOESM1_ESM.pdf]

Supplementary information for:

Aβ receptors specifically recognize molecular features displayed by fibril ends and neurotoxic oligomers

Ladan Amin[1] and David A. Harris[1*]

[1]Department of Biochemistry, Boston University School of Medicine, Boston, MA 02118

*To whom correspondence should be addressed: David A. Harris, Department of Biochemistry, Boston University School of Medicine, 72 E. Concord Street, K-225, Boston, MA 02118, USA. Email: daharris@bu.edu. Tel: 617-358-4346. Fax: 617-358-4353.

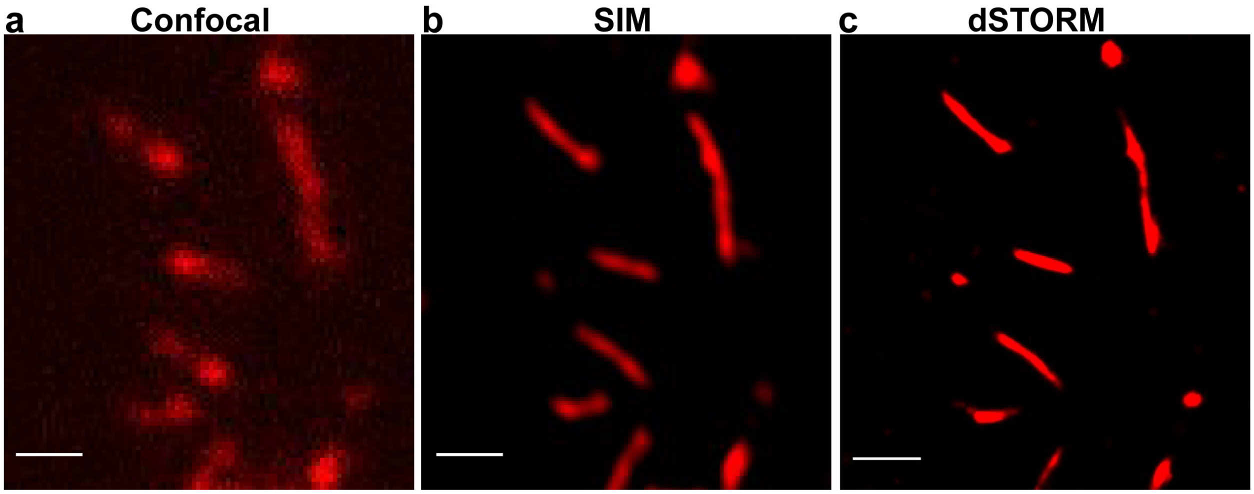

Supplementary Figure 1. Comparison of confocal and super-resolution imaging of Aβ fibrils. Images of fluorescent Aβ-Cy5 fibrils acquired by laser-scanning, confocal fluorescence microscopy **(a)**, SIM **(b)**, and dSTORM **(c)**. Scale bars are 1 μm.

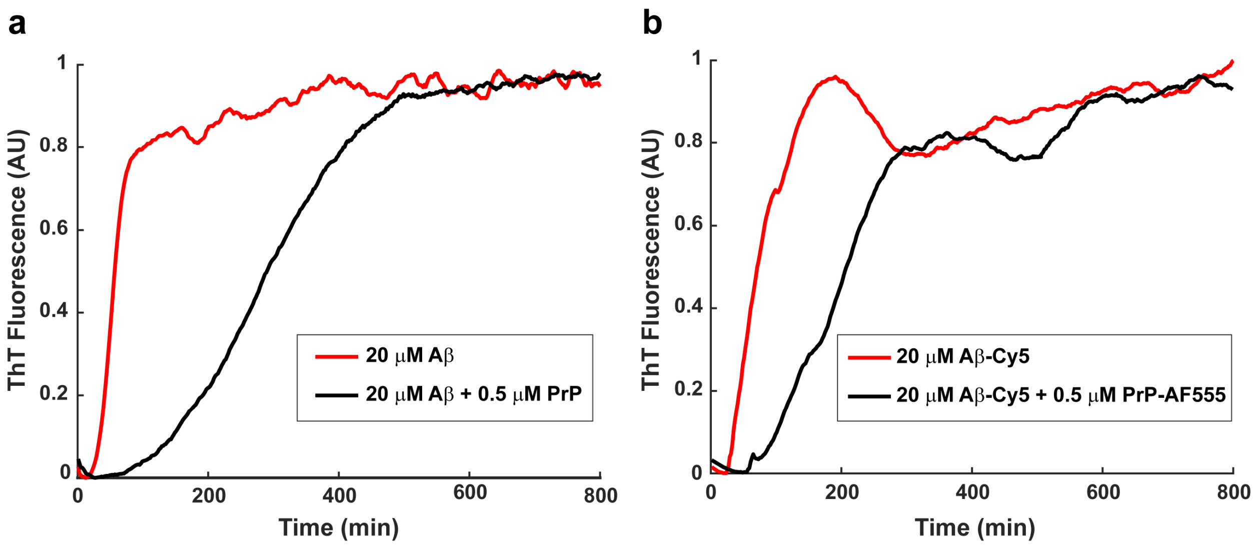

Supplementary Figure 2. Dye labeling does not affect the polymerization of Aβ or the inhibitory effect of PrP. (a) ThT curves for polymerization of unlabeled Aβ (20 μM) in the presence of 0.5 μM unlabeled PrP. **(b)** ThT curves for polymerization of 20 μM Aβ-Cy5 in the presence of 0.5 μM PrP-AF555. Each curve is the average of 3 replicates, and each condition was repeated at least 3 times.

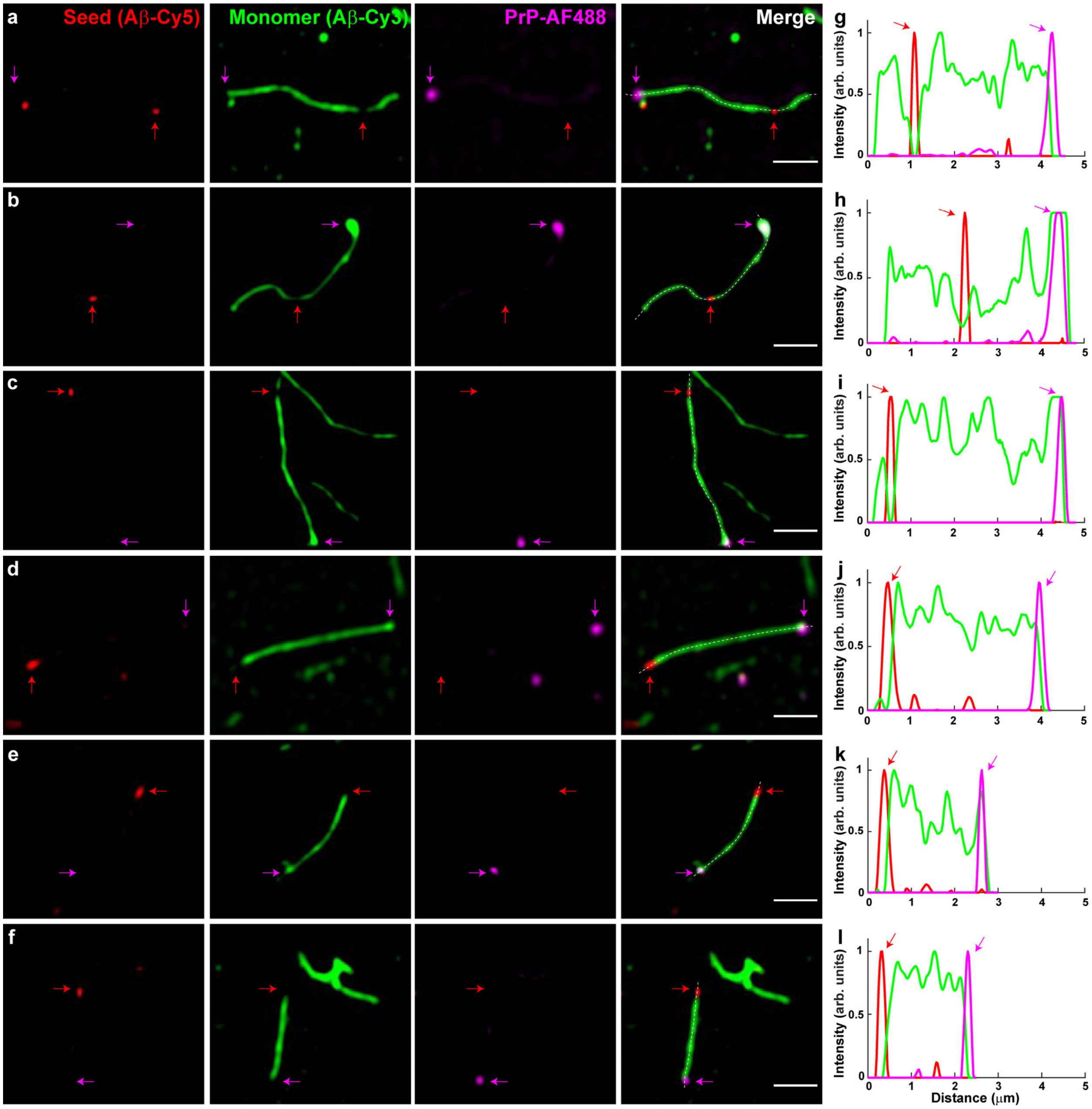

Supplementary Figure 3. PrP binds to the fast-growing end of Aβ fibrils. Preformed seeds composed of Aβ-Cy5 (red) were first incubated for 24 hrs with monomeric Aβ-Cy3 (green), to allow extension of the seeds at both ends. PrP-AF488 (magenta) was then added for 30 min, and the fibrils were imaged by three-color SIM. Panels **a-f** show six different microscopic fields containing individual Aβ fibrils, with each field imaged separately for Cy5 (Aβ seed), Cy3 (Aβ monomer), and AF488 (PrP), and the rightmost panel showing a merge of the three colors. The magenta arrow indicates PrP bound to the fast-growing end of a single fibril, represented by the long green extension from a red seed. The red arrow indicates the position of the seed. Scale bars are 1 μm. Panels **g-l** show fluorescence intensity profiles (in arbitrary units, arb. units) measured along the length of the fibrils, indicated by the white dotted lines in a-f (rightmost panels). Magenta and red arrows indicate peaks corresponding to the positions of the PrP and the seed, respectively.

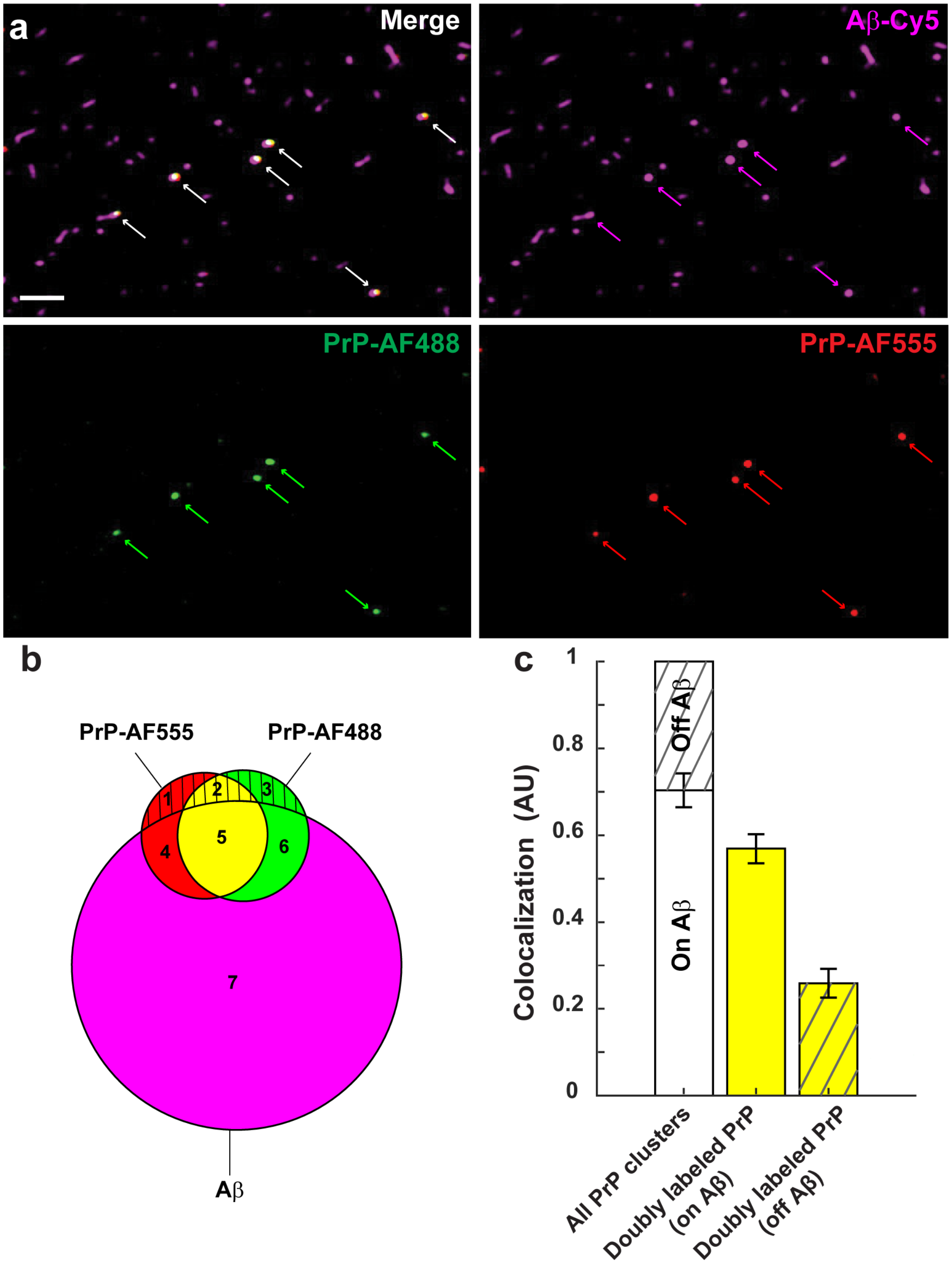

Supplementary Figure 4. More than one PrP molecule binds to each Aβ fibril end. (a) Aβ-Cy5 monomers (cyan) were incubated for 24 h in the presence of 250 nM PrP-AF488 (green) and 250 nM PrP- AF555 (red). Samples were then imaged with 3-color SIM. Arrows indicate PrP clusters containing both labels that are bound to individual Aβ fibril ends (white arrows in merged image). Scale bar, 2 μm. **(b)** Venn diagram shows the overlap of PrP clusters (AF488, green;

AF555, red; both, yellow) and Aβ-Cy5 fibrils (magenta). The numbered areas indicate categories of PrP clusters used for calculations in panel c. **(c)** Bar graph showing the proportions of all PrP clusters, either associated or not associated with Aβ fibrils (Bar #1, open and cross-hatched areas, respectively); the proportion of doubly labeled PrP clusters associated with Aβ fibrils (Bar #2, yellow); and the proportion of doubly labeled PrP clusters not associated with Aβ fibrils (Bar #3, cross-hatched yellow). The height of each bar corresponds to the numbered areas of the Venn diagram in Panel b. These values were calculated as follows, where N# represents the number of PrP clusters in each category: **Bar #1:** *On Aβ* = $(N4+N5+N6)/N_{total}$; *Off Aβ* = $(N1+N2+N3)/N_{total}$; N_{total}=N1+N2+N+N3+N4+N5+N6. **Bar #2:** N5/(N4+N5+N6); **Bar #3:** N2/(N1+N2+N3). 70±0.03% of the PrP clusters were associated with Aβ fibrils and 30±0.03% were not (Bar #1). The graph shows that 58±0.03% of the fibril-associated PrP clusters were labeled with both AF488 and AF555 dyes (Bar #2), indicating that the majority of clusters contained more than one molecule of bound PrP. In contrast, only 25±0.03% of the PrP clusters not associated with fibrils contained both labels (Bar #3), indicating that most unbound PrP molecules were monomeric.

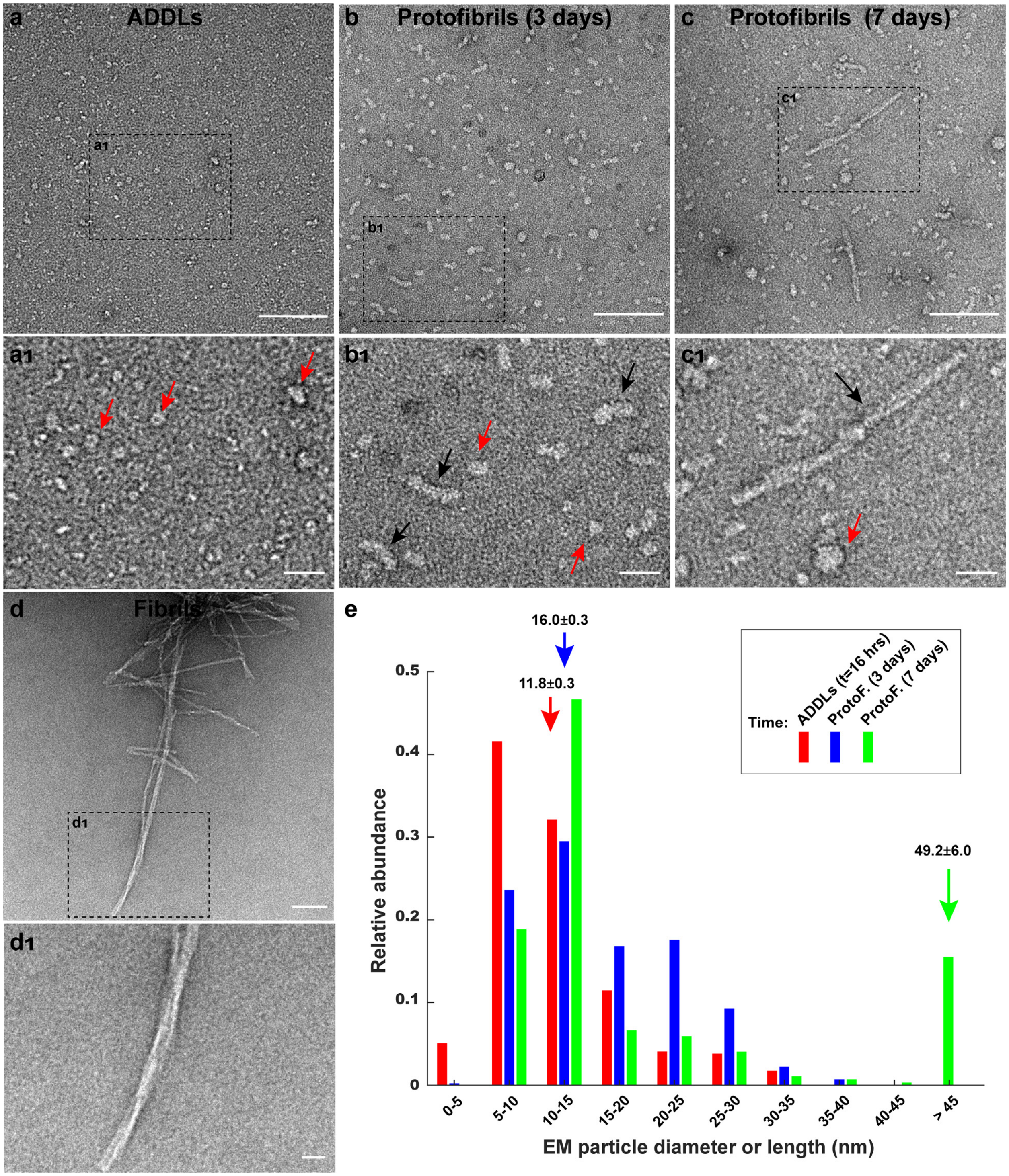

Supplementary Figure 5: Electron microscopy of ADDLs and protofibrils. (a-d) Negatively stained samples of ADDLs (16 hr) **(a)**, protofibrils (3 days) **(b)**, protofibrils (7 days) **(c)** and mature fibrils **(d)**. ADDLs were prepared by incubation of solubilized Aβ for 16 hrs, and protofibrils by incubation of ADDLs for an additional 3 or 7 days. Mature fibrils were polymerized directly from monomeric Aβ for 24 hrs. In all cases, Aβ was used at a concentration of 20 μM (monomer-

equivalents). Scale bars, 100 nm. (**a_1-d_1**) High magnification of boxed areas shown in (a-d). Red arrows in a_1-c_1 indicate globular structures and black arrows indicate short fibrillar structures. Scale bars, 30 nm. **(e)** Histogram showing the distributions of the diameters or lengths (longest dimension) of ADDLs and protofibrils measured by negative stain EM. The mean size of ADDLs (16 hrs), protofibrils (3d), and protofibrils (7d) is indicated by red, blue, and green arrows, respectively. Numbers of biological independent samples (N) and analyzed micrographs (X) in each condition are: ADDLs: N=2, X=8; Protofibrils 1: N=2, X=8; Protofibrils 2: N=2, X=14.

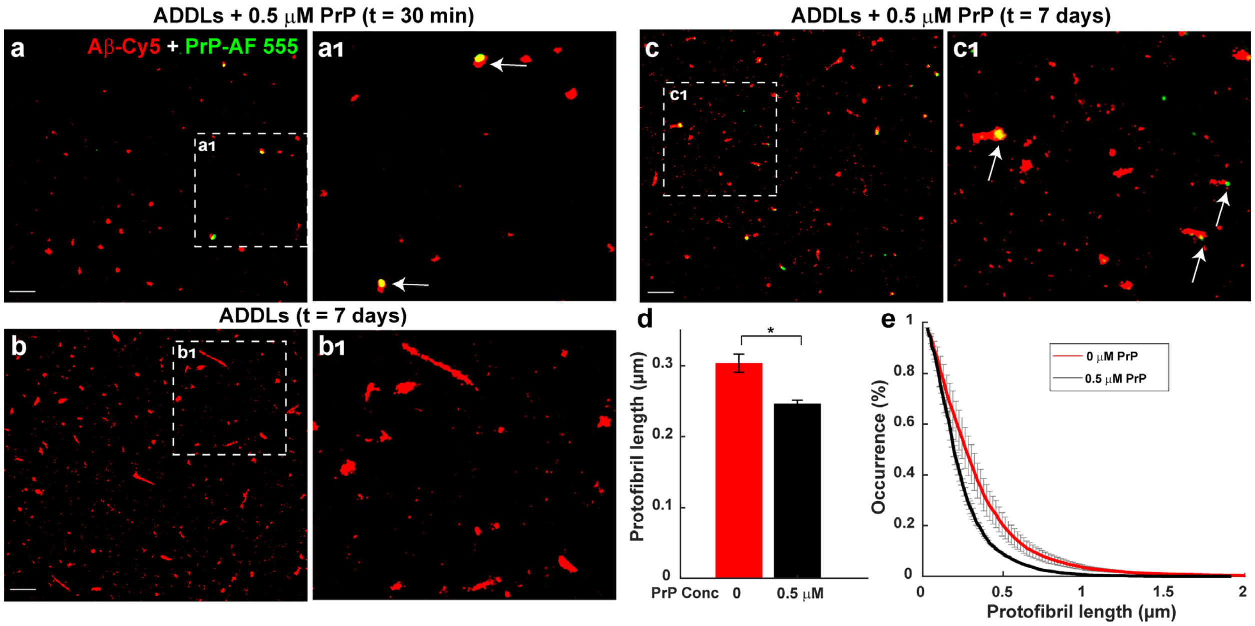

Supplementary Figure 6. PrP inhibits growth of protofibrils, and binds to one end of protofibrils. (a) Dual color dSTORM image of ADDLs prepared from Aβ-Cy5 (20 µM, red) that were incubated for 30 min with 0.5 µM PrP-AF555 (green). Panel a_1 shows the boxed area in a at higher magnification. **(b, c)** dSTORM images of protofibrils formed by incubation of ADDLs for 7 days in absence **(b)** or presence **(c)** of 0.5 µM PrP-AF555. Panels b_1 and c_1 show boxed areas in b and c, respectively, at higher magnification. Arrows in c_1 indicate the localization of PrP at the ends of the protofibrils. Scale bar, 1 µm **(d)** Bars show the mean lengths of protofibrils formed in the presence of 0 and 0.5 µM PrP**.** Data represent mean ± S.E. * P<0.05 (two-sided Student's t-test). **(e)** Cumulative distributions of protofibril lengths in the presence of 0 and 0.5 µM PrP. The number of analyzed SIM images in each condition is ≥ 9.

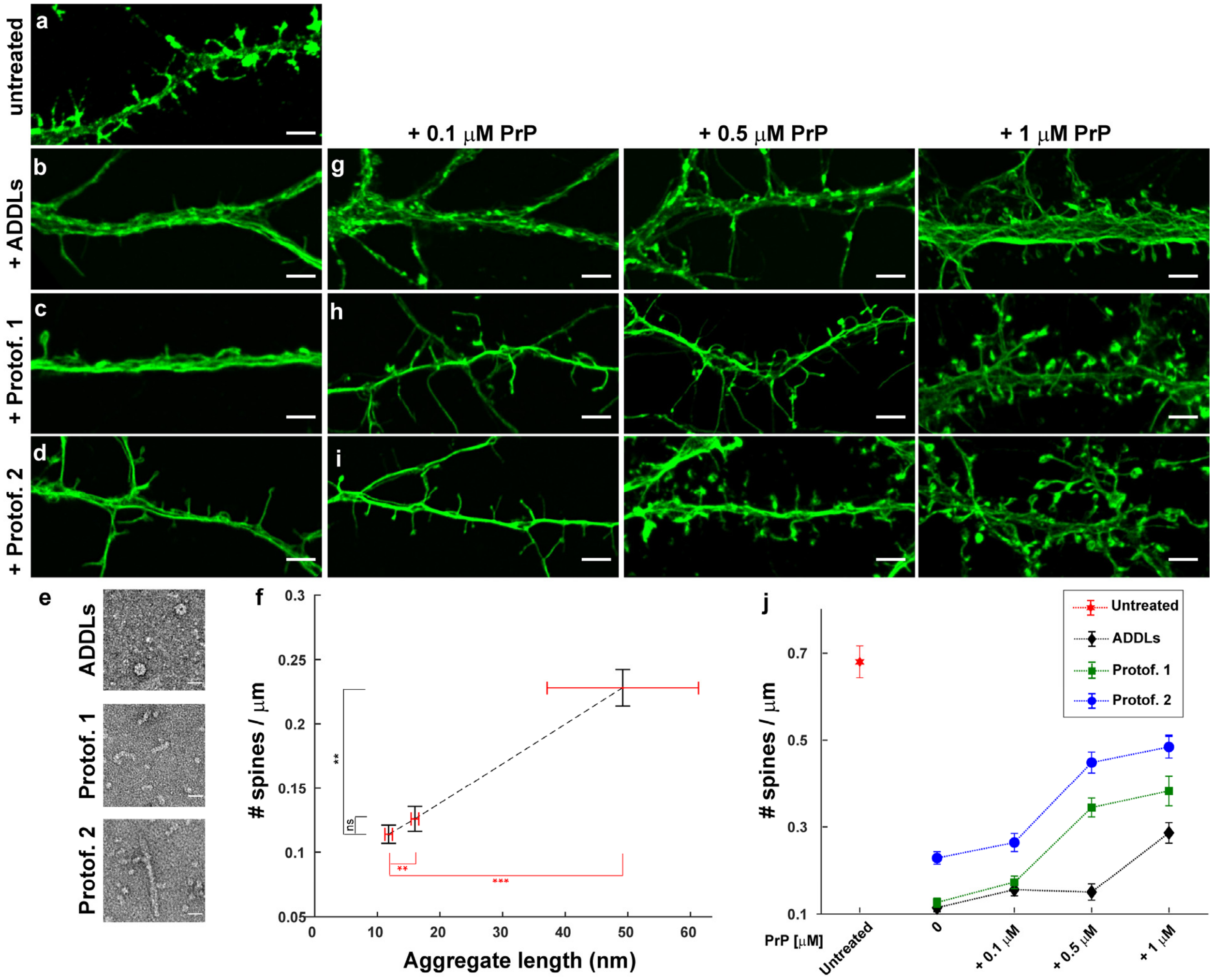

Supplementary Figure 7. Recombinant PrP reduces the neurotoxicity of ADDLs and protofibrils. Primary hippocampal neurons were untreated **(a)**, or were treated with 500 nM (monomer-equivalent concentration) of ADDLs **(b)**, protofibril preparation 1 (incubated for 3 days) **(c)**, or protofibril preparation 2 (incubated for 7 days) **(d)**. Neurons were fixed after 1 hr of treatment and stained with Alexa 488-labeled phalloidin to visualize dendritic spines. Scale bars, 2 μm. **(e)** EM images of each Aβ preparation. Scale bars, 20 nm. **(f)** Spine number per μm correlates with aggregate size. Data are presented as mean± S.E. **P<0.01 and ***P<0.001 (two-sided Student's t-test). **(g-i)** Hippocampal neurons were treated with ADDLs **(g)**, protofibrils 1 **(h)** or protofibrils 2 **(i)** that had been pre-incubated with the indicated concentrations of recombinant PrP. 20 μM ADDLs or protofibrils were pre-incubated for 10 min with 0.1, 0.5 or 1 μM of recombinant PrP. The Aβ-PrP mixture was then diluted 40-fold into neuronal culture medium to a final concertation of 500 nM Aβ equivalents. **(j)** Curves indicate the number of spines / μm on neurons treated with ADDLs and protofibrils pre-incubated with different concentrations of PrP. Error bars represent mean± S.E. The numbers of biological independent samples (N) and analyzed neurites (X) in in each condition are: N ≥ 2, X ≥ 25.

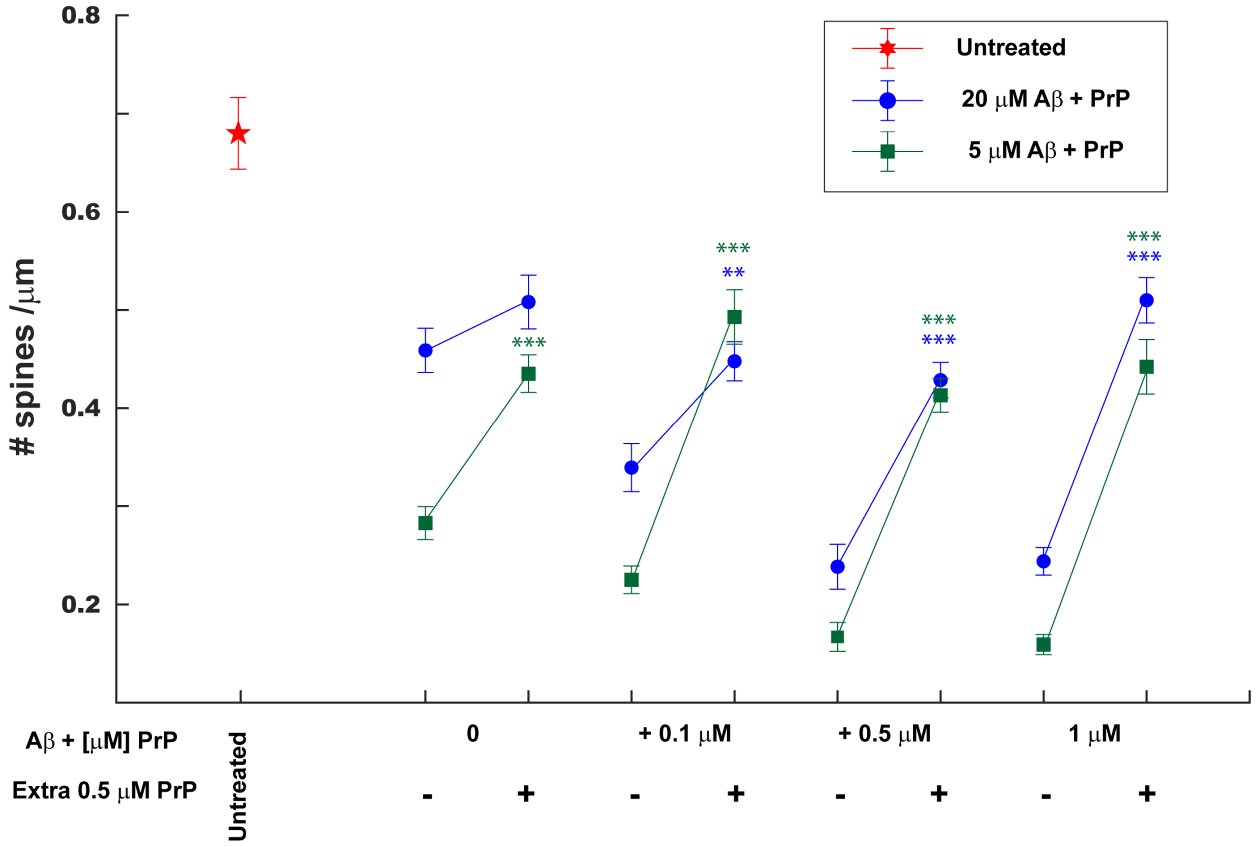

Supplementary Figure 8. Neurotoxicity assay of Aβ fibrils polymerized at two different Aβ concentrations in the presence of PrP. Aβ monomers (5 or 20 µM, blue circles and green squares, respectively) were polymerized in the presence of PrP (0, 0.1, 0.5, and 1 µM) for 24 h. The resulting fibrils were then either diluted directly into neuronal culture medium to give a final Aβ concentration of 500 nM (monomer-equivalents); or they were first incubated for 10 min with additional recombinant PrP to give a total concentration of 5 µM (equimolar to Aβ) before dilution into the culture medium (final PrP concentration of 0.5 µM). Neurons were fixed after 1 hr of treatment and stained with Alexa 488-labeled phalloidin to visualize dendritic spines. Dendritic spine number per µm is shown as mean± S.E. * P<0.05 1, ***P<0.01 1 and ***P<0.001 (two-sided Student's t-test). The red asterisk represents cultures not treated with Aβ. Numbers of biological independent samples (N) and the number of analyzed neurites (X) in in each condition are: N ≥ 2, X ≥ 36.
