# Supplementary material for: Aβ receptors specifically recognize molecular features displayed by fibril ends and neurotoxic oligomers
Source: Nat Commun. 2021 Jun 8;12:3451. doi: 10.1038/s41467-021-23507-z (PMC8187732; doi:10.1038/s41467-021-23507-z)
Supplement: Supplementary file 3 — Reporting summary [file 41467_2021_23507_MOESM3_ESM.pdf]

nature research

Corresponding author(s): David A. Harris

Last updated by author(s): 11/25/2020

Reporting Summary

Nature Research wishes to improve the reproducibility of the work that we publish. This form provides structure for consistency and transparency in reporting. For further information on Nature Research policies, see our Editorial Policies and the Editorial Policy Checklist.

Statistics

For all statistical analyses, confirm that the following items are present in the figure legend, table legend, main text, or Methods section.

n/a	Confirmed	
☐	☒	The exact sample size (*n*) for each experimental group/condition, given as a discrete number and unit of measurement
☒	☐	A statement on whether measurements were taken from distinct samples or whether the same sample was measured repeatedly
☐	☒	The statistical test(s) used AND whether they are one- or two-sided<br>*Only common tests should be described solely by name; describe more complex techniques in the Methods section.*
☒	☐	A description of all covariates tested
☐	☒	A description of any assumptions or corrections, such as tests of normality and adjustment for multiple comparisons
☐	☒	A full description of the statistical parameters including central tendency (e.g. means) or other basic estimates (e.g. regression coefficient) AND variation (e.g. standard deviation) or associated estimates of uncertainty (e.g. confidence intervals)
☐	☒	For null hypothesis testing, the test statistic (e.g. *F*, *t*, *r*) with confidence intervals, effect sizes, degrees of freedom and *P* value noted<br>*Give P values as exact values whenever suitable.*
☒	☐	For Bayesian analysis, information on the choice of priors and Markov chain Monte Carlo settings
☒	☐	For hierarchical and complex designs, identification of the appropriate level for tests and full reporting of outcomes
☒	☐	Estimates of effect sizes (e.g. Cohen's *d*, Pearson's *r*), indicating how they were calculated

Our web collection on statistics for biologists contains articles on many of the points above.

Software and code

Policy information about availability of computer code

Data collection	Super resolution and confocal image acquisition were performed with ZEN 2.3 software.
Data analysis	MATLAB R2020a was used for image analysis. A description of code has been included in Methods.<br>Image J V1.53 Software (Wayne Rayband, National Institutes of Health (NIH)) was used for processing of EM and confocal images.<br>Super-resolution images were pre-processed with ZEN 2.3 software.

For manuscripts utilizing custom algorithms or software that are central to the research but not yet described in published literature, software must be made available to editors and reviewers. We strongly encourage code deposition in a community repository (e.g. GitHub). See the Nature Research guidelines for submitting code & software for further information.

Data

Policy information about availability of data

All manuscripts must include a data availability statement. This statement should provide the following information, where applicable:

- Accession codes, unique identifiers, or web links for publicly available datasets
- A list of figures that have associated raw data
- A description of any restrictions on data availability

The datasets generated and analyzed during the current study are available from the corresponding author upon a reasonable request.

Field-specific reporting

Please select the one below that is the best fit for your research. If you are not sure, read the appropriate sections before making your selection.

- [x] Life sciences
- [ ] Behavioural & social sciences
- [ ] Ecological, evolutionary & environmental sciences

For a reference copy of the document with all sections, see nature.com/documents/nr-reporting-summary-flat.pdf

Life sciences study design

All studies must disclose on these points even when the disclosure is negative.

Sample size	The numbers of biological independent samples in each experiment is ≥ 3 unless otherwise is specified in the figure legend. Our analysis of fibril size and number included several hundred fibrils for each condition. In neurotoxicity assays, at least 25 neurites were measured in each group and experiments were repeated at least twice. Each polymerization curve was the average of at least 5 different runs.<br>No sample size calculation was performed in advance. However, the statistical analyses were performed on hundreds of individual Abeta particles in accordance with well established methods.
Data exclusions	No data were excluded
Replication	All experimental findings were reliably reproduced at least three times.
Randomization	Randomization was not applicable because we were comparing two type of fibrils where there was only a single difference between each population - i.e. the presence or absence of a receptor protein.
Blinding	Investigators were not blinded when obtaining the data but the automatic code was used to detect the size and number of fibrils therefore by definition detection was blinded.

Reporting for specific materials, systems and methods

We require information from authors about some types of materials, experimental systems and methods used in many studies. Here, indicate whether each material, system or method listed is relevant to your study. If you are not sure if a list item applies to your research, read the appropriate section before selecting a response.

Materials & experimental systems

n/a	Involved in the study	
☐	☒	Antibodies
☒	☐	Eukaryotic cell lines
☒	☐	Palaeontology and archaeology
☐	☒	Animals and other organisms
☒	☐	Human research participants
☒	☐	Clinical data
☒	☐	Dual use research of concern

Methods

n/a	Involved in the study	
☒	☐	ChIP-seq
☒	☐	Flow cytometry
☒	☐	MRI-based neuroimaging

Antibodies

Antibodies used	The following labels/antibodies were used: Alexa 488-phalloidin (ThermoFischer Scientific, Waltham, MA, Cat. #: A12379); and anti amyloid-beta antibody (6E10, mouse monoclonal primary, BioLegend, MA, Cat. #: 803001)
Validation	Antibodies were purchased commercially, and were accompanied by quality-control information from the supplier. In addition, their identity was confirmed by Western blotting or immunocytochemistry.

Animals and other organisms

Policy information about studies involving animals; ARRIVE guidelines recommended for reporting animal research

Laboratory animals	neonatal C57BL6 mice, Both-sex (P0) were used to prepare primary cultures of hippocampal neurons. All procedures involving animals were conducted according to the United States Department of Agriculture Animal Welfare Act and the National Institutes of Health Policy on Humane Care and Use of Laboratory Animals.
Wild animals	No wild animals were used
Field-collected samples	No field-collected samples were used

Ethics oversight	All procedures involving animals were conducted according to the United States Department of Agriculture Animal Welfare Act and the National Institutes of Health Policy on Humane Care and Use of Laboratory Animals. Hippocampal neurons were cultured from P0 pups as described.

Note that full information on the approval of the study protocol must also be provided in the manuscript.
